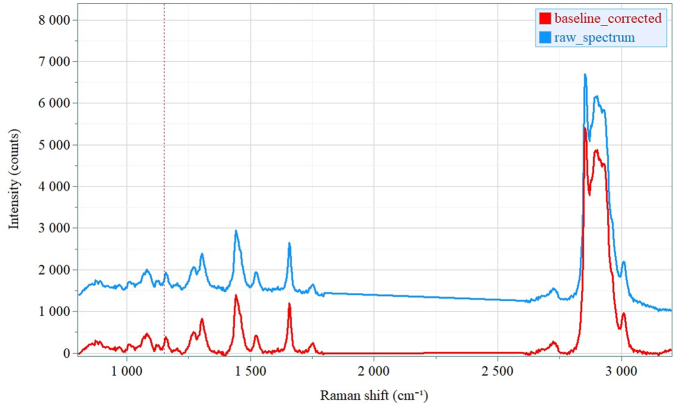# Raman analysis of breast cancer-associated adipocytes: A chemometric pipeline for lipid biochemistry profiling

**DOI:** 10.1016/j.jlr.2026.101046

**Published:** 2026-04-20

**Authors:** Pooja Girish, Pascaline Bouzy, Emilie Buache, Catherine Muller, Charlotte Vaysse, Landry Blanc, Sebastien Legendre, Olivier Piot

**Affiliations:** 1 HORIBA FRANCE SAS, Loos, France; 2 BioSpecT Unit, UR 7506, University of Reims Champagne-Ardenne, Reims, France; 3 Institut de Pharmacologie et de Biologie Structurale (IPBS), CNRS, Université de Toulouse, Université Toulouse III - Paul Sabatier (UPS), Toulouse, France; 4 Département de Chirurgie Gynécologique Oncologique, CHU-Toulouse, Institut Universitaire du Cancer de Toulouse-Oncopole, Toulouse, France

**Keywords:** Breast Cancer, Cancer Associated Adipocytes (CAAs), Obesity, Raman Spectroscopy, Data processing, chemometric pipeline

## Abstract

This study describes an integrated chemometric pipeline to analyse Raman spectra from breast tissue adipocytes, distinguishing Cancer associated adipocytes (CAAs) from normal adipocytes (NAs) and assessing the impact of obesity. Raman spectra were acquired from NAs and CAAs from the invasive front of breast tumor in 10 patients (5 normal weight, NW; 5 obese weight, OW). Extended Multiplicative Scatter Correction (EMSC) was adapted to correct carotenoid spectral interference. Random forest (RF) classifier was used for identifying discriminant wavenumbers and Uniform Manifold Approximation and Projection (UMAP) for visualization, with clustering quality assessed using silhouette scores. The results show the effectiveness of the pipeline in correcting the interferences and in identifying the key discriminant spectral regions. Informative wavenumbers highlighted differences in lipid unsaturation (C=C stretch at 1,655 cm^−1^, =C-H stretching at 3,010 cm^−1^), triglyceride composition (C=O stretching at 1745 cm^−1^) and chain packing (CH_2_ stretching 2,840–2,880 cm^−1^), revealing greater biochemical heterogeneity in CAAs. In summary, this integrative approach of data processing and analysing provides an effective framework for studying subtle spectral differences in samples. The pipeline successfully distinguished CAA and NA phenotypes, establishing a foundation for identifying spectroscopic biomarkers of adipocyte pathological remodelling in breast cancer.

Lipid molecules are not just cellular fuel, but they play a multifaceted role in tumor progression, by supporting cancer cell growth, survival, metastasis and immune evasion through metabolic reprogramming and interaction with the tumor microenvironment (1, 2). Raman spectroscopy has emerged as an analytical tool with high potential for probing these molecules, as the technique is non-invasive and label-free, allowing the characterization of lipids by their unique vibrational bands (3). It leverages on these vibrations, particularly the C-H and the C-C bonds abundant in lipids, which act as molecular fingerprints, revealing qualitative and quantitative details about the biochemical properties. The early studies identified the key spectral features such as the CH_2_ scissoring band at 1,440 cm^−1^, the C=C stretch at 1,655 cm^−1^ and the C=O at 1745 cm^−1^ (4). And recent studies have demonstrated the application of Raman spectroscopy for investigating lipid metabolism across diverse biological systems, from immune cell activation to cancer progression (3, 5). Advanced chemometric approaches such as Principal Component Analysis (PCA) and Partial Least Squares Discriminant Analysis (PLS-DA) have proven essential for extracting meaningful information from the complex spectral data (3). This information allowed researchers to quantify lipids in human-derived cell lines with remarkable precision. But as the samples shift towards complex biological tissue samples the limitations of these traditional approaches became starkly apparent.

One of the compelling and challenging applications of Raman lies in studying the Cancer Associated Adipocytes (CAAs). These cells, found at the tumor invasive front, undergo profound pathological reprogramming and exhibit tumor-supportive phenotype characterized by altered, proinflammatory secretory profiles and metabolic activity. These cells mediate the bidirectional signalling between the normal adipocytes, which are the precursors of CAAs, and the tumor cells to sustain tumor progression (6). This metabolic crosstalk is further exacerbated by systemic metabolic dysregulations, particularly obesity (7). Obesity exerts a profound influence on CAAs, driving alterations in their phenotype and function that facilitate breast tumor progression. A key aspect of this interaction lies in metabolic crosstalk, whereby CAAs deliver lipids to cancer cells through enhanced lipolysis and fatty acid transfer. Notably, obesity amplifies this lipid supply, thereby reinforcing the metabolic support that CAAs provide to malignant cells.

Raman spectroscopy has identified the unsaturation level in adipocytes, revealing how CAA supplies energy-rich lipids to their adjacent tumor cell (8). The technique has also succeeded in identifying spectral biomarkers that distinguish CAAs from normal adipocytes in in vitro studies, by the shifts in the Raman bands related to lipid molecules. Hence, this could provide insights into lipid metabolic alteration during the transition from normal adipocytes to CAAs (9).

Recent studies of human breast tissues have confirmed that Raman spectroscopy, coupled with machine learning, can detect CAAs in clinical breast tissue samples and differentiate them from normal adipocytes via lipid-associated shifts. However, the results of these studies are constrained by significant methodological challenges such as the dependency on high signal to noise ratios and spectral mixing (10). Our work presents a reworked analytical workflow to unravel biochemically relevant variabilities, such as those linked to adipocyte transformation, impact of obesity on lipid profiles but also non-informative variations such as carotenoid interference. Finally moving beyond detection, we confront the challenge of translating complex spectral patterns into interpretable lipid metrics, enabling the investigation and understanding of clinically relevant factors.

Our work addresses these challenges by integrating UMAP and Random Forests (RF) to analyze single-cell Raman spectra from adipocytes and CAAs. While studies combining UMAP with RF have been tested for pathological classifications and tissue characterization, this synergy remains untested for Raman-based CAAs studies. RF's resilience to noise and collinearity complements UMAP's ability to untangle non-linear trajectories is a perfect match for heterogeneous spectral data (11). The quality of the clusters produced by UMAP was quantitatively assessed by calculating the silhouette scores (12). Our approach can be applied to identify a concise set of lipid biomarkers that track CAAs evolution from the normal adipocyte phenotype to the pathological state.

## Materials and Methods

### Tissue sample acquisition

In this study, 20 samples of human breast tissues from 10 individuals were investigated using Raman spectroscopy. Two different types of tissues were collected from each individual; one type of tissue corresponded to the normal mammary adipose tissue (NA) while the other was the cancer-associated adipose tissue (CAA) from the invasive front of the tumor. Among the 10 individuals, 5 patients fell under the category of obese weight (OW), and the other 5 patients were normal or lean weight (NW). The obese and normal/lean weight were calculated based on the body mass index of each subject. The samples were obtained from patients undergoing mastectomy for breast cancer at the IUCT-O (Cancer Centre of Toulouse – Oncopôle). These samples have been reclassified for medical use. In accordance with French law, these collections are declared and authorized by the Ministry of Higher Education and Research (DC-2020-4074; AC-2020-4031). The Biologic Research Center undertakes to comply with data processing in accordance with reference methodology MR004. Patient information notices and the patient non-objection form for the use of samples for research comply with GDPR (General Data Protection Regulation) requirements. This study was performed according to the latest revision guidelines of the Helsinki Declaration.

Tissue samples were preserved at −80°C and later sectioned in a cryotome, which was set to −34°C to ensure sectioning of quality without delocalization of the lipid. Then, 20 μm-thick sections were deposited on CaF_2_ glass slides. These sections were stored at 4°C in between analytical measurements. The sample details are presented in Table 1.

**Table 1:** Sample details

Sample name	Age	BMI (Kg/m^2^)
NW1	70	22.2
NW2	50	21.88
NW3	81	22.03
NW4	65	21.78
NW5	62	19.81
OW1	50	30.078
OW2	68	31.8
OW3	71	32
OW4	55	30.78
OW5	66	31.25

### Raman spectral acquisition

The Raman spectra from the sample were acquired using a confocal microscope, LabRAM Soleil of HORIBA. The Labspec 6 spectroscopy suite functions as the software interface for the microscope. Initial optimization of the acquisition parameters for the confocal Raman microscope was performed to obtain high-resolution data. The laser excitation parameters were selected to 532 nm for the wavelength and 80 mW for the power, but the power at the sample was controlled by neutral density (ND) filter at 20% to avoid any damage to the sample. The 532 nm wavelength is particularly effective for Raman spectral acquisition of biological samples, it achieves a compromise between high scattering intensity and limited fluorescence, while allowing optimal CCD sensitivity on the entire spectral range even in the high wavenumber region. The confocal hole was opened to a diameter of 200 microns. The spectral range of measurement was divided into 2 windows with the fingerprint region from the wavenumber 800- 1800 cm^−1^ and the high wavenumber region from 2,500 to 3,100 cm^−1^, avoiding thus measurement in the silent region and consequently reducing the overall time for acquisition. A 1200 lines/mm grating was chosen to spectrally disperse the signal to the detector. From the sample sections, areas of 30 μm × 30 μm were selected as regions of interest (ROI) for mapping. A 100x objective with NA 0.9 was used to focus the laser beam on to the sample and to collect the back-scattered photons. A motorized sample stage was used for the acquisition of spectra from the ROI through mapping with a step size of 1 μm in X and Y directions. The acquisition time at one pixel was fixed to 10 s and with 2 accumulations. Multiple accumulations were used to remove spikes from the cosmic rays. From each ROI, 961 spectra were collected through the mapping. The acquired spectra were then pre-processed in Labspec 6 software (Version 6.7.2) as follows: (1) baseline correction using n^th^ order polynomial, (Supplemental Fig. S1) (2) normalization using the standard normal variate, (3) smoothing using Savitzky-Golay algorithm with a polynomial order of 2, which removes local signal noise while preserving the shape of the signal, (4) then the spectral baseline is were shifted back to zero intensity. After the pre-processing all the datasets are concatenated together into one dataset with the sample names as labels.

### Data processing

#### Extended multiplicative scatter correction (EMSC) for interference correction

EMSC models an experimental spectrum as a combination of a reference spectrum (also called target spectrum), a set of polynomial terms for baseline correction and one or more interference spectra. The algorithm does not remove the spectral interferences but neutralize their effect.

The tissue samples contained carotenoids in variable concentration, generating signals that can be intense due to Raman resonance scattering effect. First, differences between patients are due to diet. In addition, intensity variations can be observed within the spectral mapping of one ROI because of temperature dependence of resonance Raman signals (13).

**Fig. 1:**
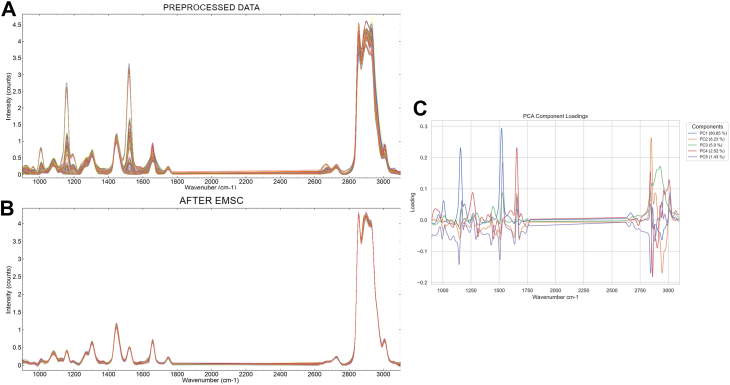
A: concatenated data before EMSC, B: data after EMSC, C: PC loadings used as interference for EMSC.

Intensity variations of carotenoids peaks cannot be normalized using the preprocessing techniques described above. This interference due to temperature dependence of resonance Raman spectra of carotenoid arises because of laser wavelength used in the spectral acquisition being close to the resonant absorption wavelength of carotenoids. Hence, an additional preprocessing step was added to refine this interference correction by modelling spectral variability associated with carotenoids, using loadings of a principal component analysis (PCA) as inputs of the EMSC interference matrix. This was implemented in Quasar (Version 1.11.1). PCA was applied to the pre-processed data to extract the principal components with dominant variations in the peaks. The 5 components explained more than 96% of the variance. Here in this experiment, we have used the average of the datasets as the reference spectrum, the polynomial order of 4, and the principal components from the PCA of the pre-processed data as the interference spectra. Figure 1 depicts the concatenated spectra from all the sample sets before (Fig. 1A) and after (Fig. 1B) the EMSC correction, along with the PC components used as the interference spectra (Fig. 1C).

### Data analysis

The dataset was divided into different subsets as listed in Table 2. The spectra were then cut into the fingerprint region and the high wavenumber region, between 900–1800 cm^−1^ and 2,700–3,100 cm^−1^ respectively, omitting the silent region for the analysis. All the subsequent treatments were carried out separately in each of these 2 spectral regions. The discriminant wavenumbers distinguishing the different categories were identified using Random Forest (RF) classifier implemented in Python's scikit-library (Version 1.3.0). An ensemble of 100 decision trees was trained with Gini impurity (default) as the splitting criterion and 10-fold cross validation was applied to mitigate overfitting. To ensure reproducibility, the random state was fixed (seed = 42), and the class imbalance was addressed by stratifying sampling during tree construction. The discriminant wavenumbers were ranked based on the feature-importance, and the top 15 features were selected for further downstream analysis. Uniform Manifold Approximation and Projection (UMAP) (Python UMAP-learn version 0.5.3) was used for the dimensionality reduction and visualization of the data distribution. To ensure robustness, embeddings were initialized using spectral decomposition and repeated across 10 random seed (42–51), with the most stable projection retained. UMAP outputs were visualized as 2D scatterplots. To quantitatively assess the clustering and separability of the different categories of data studied, silhouette scores were calculated in addition to qualitative inspection of spatial overlap.

**Table 2:** Distribution of data for analysis

Data	Data (NW)	Data(OW)
20 datasets	10 datasets	10 Datasets
Between NA & CAA	Between NA & CAA	Between NA & CAA

## Results

### Data partitioning and unsupervised analysis of spectral data using UMAP

**Fig. 2:**
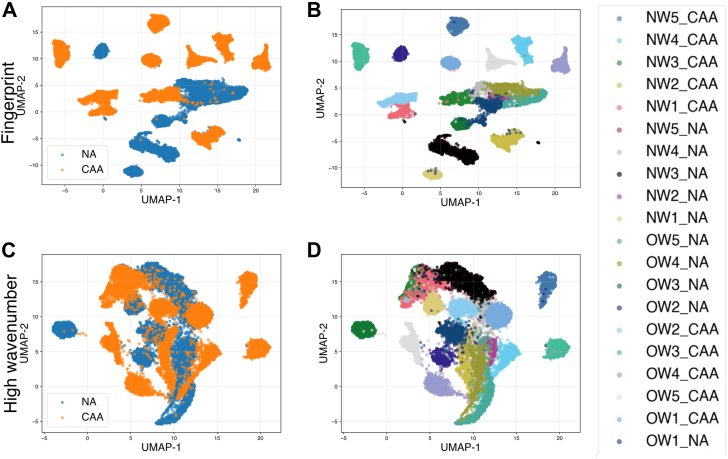
UMAP of the data between CAA and NA on all wavenumbers: (A) and (B) for the fingerprint region, (C) and (D) for the high wavenumber region.

**Fig. 3:**
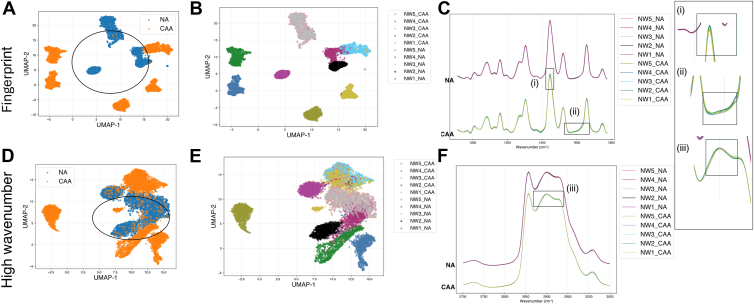
UMAP of the dataset of NW samples between CAA and NA on all wavenumbers (A) and (B) for the fingerprint region, (D) and (E) for the high wavenumber region. (C) and (F) is the mean spectra of the NA dataset compared to that of the CAA datasets for the fingerprint region and the high wavenumber region, respectively. (in inserts (i), (ii) and (iii): highlighted areas as marked in the mean spectra.

**Fig. 4:**
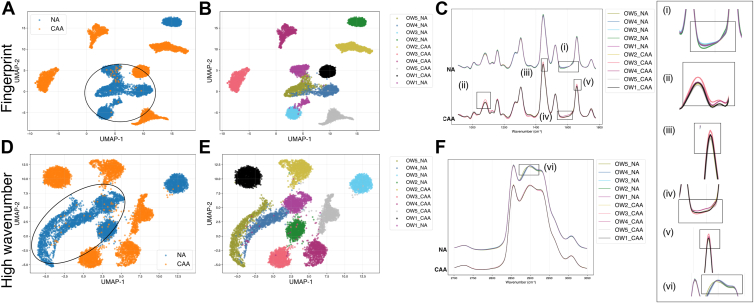
UMAP of the dataset of OW samples between CAA and NA on all wavenumbers (A) and (B) for the fingerprint region, (D) and (E) for the high wavenumber region. (C) and (F) is the mean spectra of NA dataset compared to that of the CAA datasets for the fingerprint region and the high wavenumber region, respectively. (in inserts (i), (ii), (iii), (iv), (v) and (vi): Highlighted regions as marked in the mean spectra.

Table 2 presents the specifics regarding how the data was partitioned for different analysis. The preliminary experiment aimed to investigate total datasets from the 20 samples and find the relative distribution of the NA and CAA in a reduced-dimensional space. As mentioned in the materials and methods, the wavenumber range is divided into fingerprint region and high-wavenumber region while acquisition of the spectra; consequently, the analysis is also performed for these wavenumber ranges independently for finding the discriminant wavenumbers. This allows us to assess which spectral region is the most informative about the biochemical differences between CAA and NA. Uniform Manifold Approximation and Projection (UMAP) was employed to visualize how the Raman spectra of CAA and NA were distributed. The blue scatter points represented NA, while the orange ones denoted the CAA dataset. In addition, silhouette scores were calculated from the UMAP projections to provide a metric for an objective evaluation of the dataset's separability. For both spectral regions, CAA and NA data were dispersed along the 2 dimensions of the UMAP plot without clustering for the target category (CAA or NA). As it can be observed in the Fig. 2B, D, the UMAP projection preserved the individual sampling, especially for CAA, with compact clusters associated with the different samples. In the initial analysis, NW and OW samples were collected together, which may have resulted in BMI affecting the spectral profiles of CAA and NA, complicating the identification of Raman features specific to each adipocyte type. Therefore, subsequent experiments analyzed NW and OW samples separately. Figures 3 and 4 depicts the UMAP projections of CAA and NA spectra, for NW and OW samples, respectively. Concerning the NW samples, the NA spectra of the different samples appeared quite grouped at the center of the projection, as underlined by a black circle on Fig. 3A corresponding to the fingerprint region. The CAA data were separated from the NA but dispersed over the 2D UMAP plot, reflecting more biochemical variability for CAA than for NA. For the high wavenumber region, similar tendency can be drawn, for NA as circled in Fig. 3D, but with less clear separation between NA and CAA. From Fig. 3D, we were able to identify CAA spectra of one sample, NW5_CAA, far from the rest of the dataset. To explore the observed separation, we calculated the mean spectra of all samples (Fig. 3C, F) and compared them to determine whether this sample exhibited any notable deviation. And it has been found that the mean spectrum of this sample presents very subtle differences in certain spectral regions, which are highlighted in the figure insert (i), (ii), and (iii).

Similarly, Fig. 4 is the presentation of the OW samples. In Fig. 4, the cluster formation of the NA datasets is marked in circles (Fig. 4A, D), In the UMAP of the fingerprint region, among NA, OW2_NA showcases a distinct cluster away from the main cluster group of NA (Fig. 4B). The mean spectrum calculation shows some subtle deviations in mean of this sample from the rest of the sample Fig. 4C (insert (i)). In the UMAP of the high-wavenumber region, a similar separation from the main cluster of the NA is shown by OW2_NA; the mean spectra of this sample can be observed slightly deviating from the rest of the spectra-Fig. 4F (insert (vi)). This could explain the separation of these samples from other NA sample clusters. In the mean spectra of CAA in the fingerprint region, the mean spectrum of OW3_CAA showcases similar spectral differences Fig. 4C, (insert (ii), (iii), (iv) and (v)). This could explain why the cluster of this sample is located at a distance from all the other clusters.

### Supervised identification of discriminant wavenumber and silhouette score

**Fig. 5:**
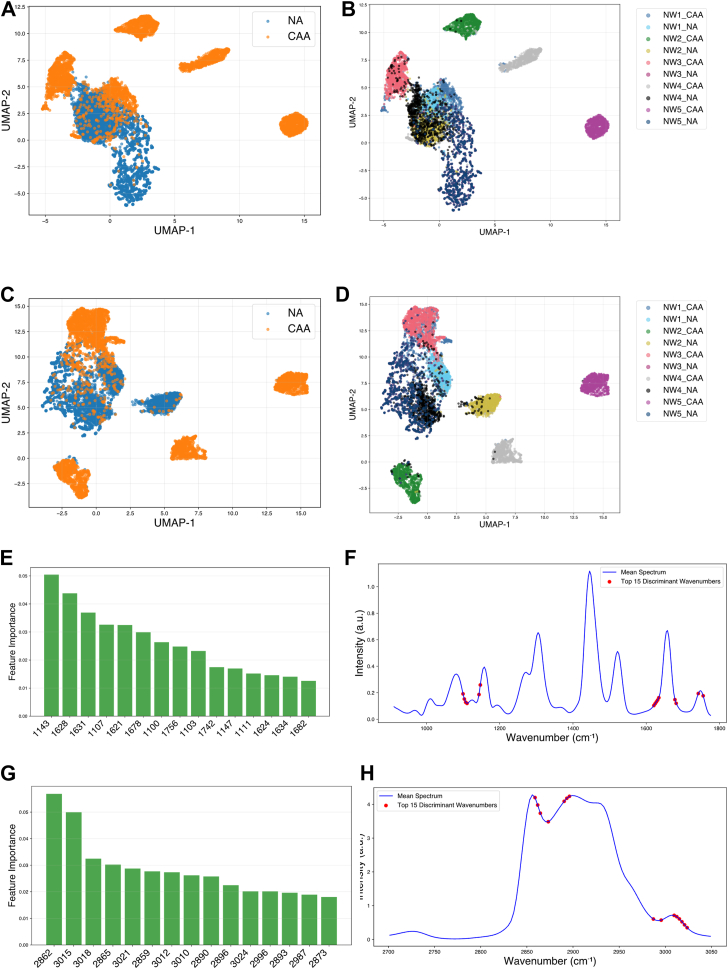
UMAP of the top 15 discriminant wavenumbers after the random forest feature selection between CAA and NA for NW dataset (A) and (B) for the fingerprint region, (C) and (D) for the high wavenumber region. Top 15 discriminant wavenumbers selected by random forest classifier between CAA and NA for NW dataset, ranked based on the feature importance (E) in the fingerprint region (G) in the high wavenumber region. Precise location of the discriminant wavenumbers in the mean spectra is depicted in the (F) for the fingerprint region and (H) for the high wavenumber region.

**Fig. 6:**
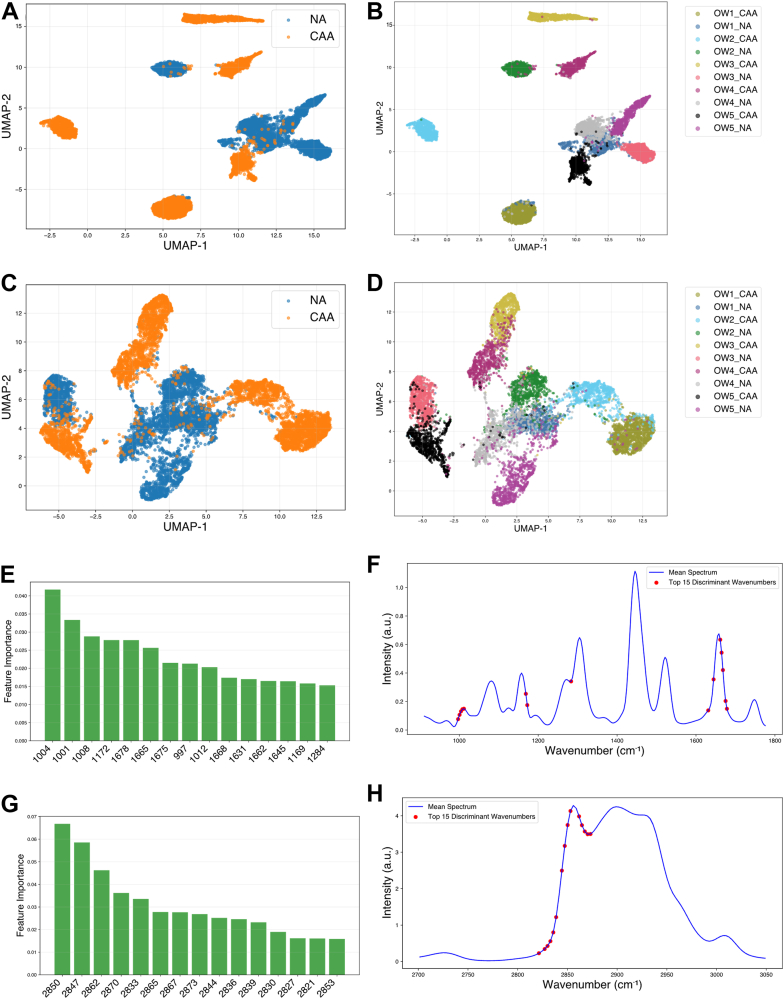
UMAP of the top 15 discriminant wavenumber after the random forest feature selection between CAA and NA for OW dataset (A) and (B) for the fingerprint region, (C) and (D) for the high wavenumber region. Top 15 discriminant wavenumbers selected by random forest classifier between CAA and NA in the normal weight (NW) dataset, ranked based on the feature importance (E) in the fingerprint region (G) in the high wavenumber region. location of the discriminant wavenumbers in the mean spectra is depicted in the (F) for the fingerprint region and (H) for the high wavenumber region.

For a more precise identification of Raman features involved in the distinction between CAA and NA, a discriminant wavenumbers selection was tested using a supervised approach for each of the 2 categories of samples, namely OW and NW. Here, a Random Forest classifier was employed. The discriminant wavenumbers found by the algorithm were ranked based on their feature importance and the selected top 15 wavenumbers are displayed in Fig. 5E–H for NW datasets and in Fig. 6E–H for OW datasets. The results are depicted in Fig. 5A–D for the NW dataset and in Fig. 6A–D for OW dataset. Revisiting the UMAP analysis across the entire wavenumber range for the fingerprint region in Fig. 3A, B for NW, reveals that while each sample forms tight and coherent clusters, NA and CAA do not separate into distinct clusters. However, in the high-wavenumber region in Fig. 3C, D, there are partial but distinct clusters formed by the NA and CAA datasets. The silhouette score for this UMAP scatterplot, without feature selection, was calculated to be 0.20 for the fingerprint region and 0.10 for the high-wavenumber region. The results demonstrate significant improvement following feature selection and the application of UMAP to the discriminant wavenumbers identified by the random forest algorithm. In Fig. 5A, B, the UMAP shows tighter clustering within NA and CAA datasets in the fingerprint region with a calculated silhouette score of 0.27, while in high-wavenumber region, in Fig. 5C, D, similar embedding is observed with NA datasets forming tighter cluster with some scattered clusters of the CAA in the space with a silhouette score of 0.16 for this distribution. In the analysis of the OW datasets, UMAP before feature selection (Fig. 4A, B) for the fingerprint region shows weaker clustering of the 2 categories with a silhouette score of 0.09, which increased to 0.20 following feature selection (Fig. 6A, B). While in Fig. 4D, E, NA forms one large cluster along with additional smaller clusters, whereas the CAA was largely distributed in more compact, distinct clusters, with a silhouette score of 0.09. In Fig. 6C, feature selection enhanced the silhouette score to 0.16, and the NA cluster showed a more compact distribution with CAA clusters distributed around it. The discriminant wavenumber selection using RF classifier has been shown to enhance the performance of UMAP, distinguishing between the categories of the datasets.

The major wavenumber regions selected by the algorithm in the NW dataset in Fig. 5E–H, were 1,143 cm^−1^ near the 1,155 cm^−1^ peak assigned to the carotenoid, 1,628–1,682 cm^−1^ assigned to the C=C stretching of lipid and amide I of protein along with 1747 cm^−1^ and 1756 cm^−1^ which are part of the peak assigned to C=O stretching of lipid ester (14). In the high-wavenumber region the top-ranked discriminant wavenumbers corresponded to the stretching of C-H associated with the C=C bonds around 3,009 cm^−1^. The other wavenumbers were distributed across the 2,830–3,000 cm^−1^ region. This region corresponds to the C-H stretching vibrations of the CH_2_ groups in lipid hydrocarbon chains. This region serves as a sensitive region for lipid chain conformation, packing density and structural order. The peak around 2,840 cm^−1^ is assigned to the symmetric CH_2_ stretching mode, while the peak at 2,880 cm^−1^ corresponds to the asymmetric CH_2_ stretching mode. As shown in Fig. 6E, for the OW datasets, the discriminant wavenumbers in the fingerprint region exhibit a trend consistent with the previous experiments. Specifically, wavenumbers in the range of 1,640–1,680 cm^−1^ and 1,169 cm^−1^–1,172 cm^−1^ are identified, with an addition of wavenumbers in the range of 1,000–1,012 cm^−1^. The peaks in this region correspond to CH_3_ rocking coupled with the C-C stretching of carotenoids. In Fig. 6G, H, the major discriminant wavenumbers are distributed in the region of 2,830–2,870 cm^−1^, where the peaks are assigned to the symmetric and asymmetric stretch of lipids.

## Discussion

The objective of this study was to determine an optimal processing and analysis technique for Raman spectral data of lipids from breast adipose tissue. We integrated the Random Forest algorithm with UMAP for this purpose. To the best of our knowledge, this study represents the first application of such an integrative approach to analyze lipid Raman signals from adipocytes. The important findings of this work are as follows: the preprocessing of the data was accompanied by an additional step for the correction of interference signals from the carotenoid content in certain samples. This interference signal arises from the temperature dependence of the resonance Raman signal of the carotenoid molecule. To address this issue, we developed a method that combines Extended Multiplicative Scatter Correction (EMSC) with Principal Component Analysis (PCA). The principal components obtained from the analysis were used as interference spectra for EMSC. This correction was applied prior to further analysis of the Raman spectra with the Random Forest algorithm, which identified discriminant wavenumbers that differentiate the Raman spectral data between categories in the samples based on feature importance ranking. The algorithm has found in a few instances where the wavenumbers corresponding to carotenoids are discriminant, especially in the wavenumber regions between 1,100 and 1,180 cm^−1^, which could suggest that when the interference due to carotenoid intensity is corrected, the algorithm was able to pick up the difference in the carotenoid composition or the quantity between the samples. The initial experiment (Fig. 2) focused on investigating global data from 20 different samples from different adipocyte locations and with different body mass indices. The objective of this study was to identify spectral features that can distinguish normal adipocytes (NA) from cancer-associated adipocytes (CAAs). There have been studies that states that CAAs have different morphology and composition in comparison with NA (2). They also exhibit different metabolic activity and secretory profile, even driving chronic inflammation, fostering a tumor-promoting microenvironment (3). Raman spectroscopic studies have revealed frequent display of unsaturated lipid profiles by CAAs, where the unsaturation is quantitatively evaluated by the ratio of Raman peak intensities (15). Raman studies have also demonstrated shifts in lipid unsaturation in obesity and related metabolic diseases (4).The spectral differences are very subtle and hence the use of multivariate statistical analysis is required to highlight the discriminant signals between the sample sets. This experiment was repeated after isolating the normal weight and obese weight samples separately.

The discriminant wavenumbers found by the algorithm are listed in Table 3. These following discriminant wavenumbers are common for all the 3 experiments mentioned above, 1,001–1,008 cm^−1^ a peak in this area can correspond to phenylalanine, or CH_3_ rocking coupled with C-C stretching of carotenoid, 1,107-1,176 cm^−1^ a peak in this region is assigned to C-C stretch of lipids, proteins and carotenoids, 1,620–1,680 cm^−1^ corresponding to C=O stretching of amide or C=C stretching of fatty acids, 2,820–2,890 cm^−1^ corresponding to the symmetric and asymmetric stretch of C-H bond of lipids, 3,010-3024 C-H lipid stretching. The other wavenumbers that have appeared in any one of the experiments are: 1,337–1,347 cm^−1^ vibrations in collagen, 1740–1756 cm^−1^ C=O stretch of lipids. (1) These wavenumbers indicate potential differences in lipid saturation, oxidation, or triglyceride composition between the 2 categories. The UMAP on the top 15 wavenumbers selected by the algorithm showed comparatively improved results for the 3 experiments with compact and coherent clustering of the NA and CAA datasets. To further investigate the impact of the body mass index on lipid composition, 2 more analyses were performed isolating the NA and CAA datasets. The impact of obesity on normal adipocytes has been studied extensively, whereas its effect on CAAs is relatively underexplored. Obesity has been demonstrated to markedly impact the structure, function, and molecular signalling pathways in normal breast adipocytes, thereby fostering a microenvironment conducive to tumor development (4). The most relevant wavenumbers identified by the algorithm suggest different biochemical states for CAA from NA. From Table 3, the wavenumbers can be correlated to differences in unsaturation degree and lipid storage. In obese conditions the lipids have been shown to have lower degree of unsaturation in the normal adipose tissues, reflecting lower proportion of polyunsaturated fatty acids (16). This aligns with the studies which have shown that obesity promotes increased saturated lipid accumulation (17). Raman spectroscopy has revealed the increase in lipid accumulation in obesity along with notable differences in protein to lipid ratio, indicating the triglyceride storage outpaces collagen buildup (18). These findings collectively demonstrate the capacity of Raman analysis for identifying the remodelling of adipose tissue in different pathological states, where the biochemical alterations serve as the spectroscopic signatures marking the transition from healthy to pathological state.

**Table 3:** Wavenumbers selected by RF algorithm

Common range of the spectral feature selected by RF (cm^−1^)	Discriminant wavenumbers in NW (cm^−1^)	Discriminant wavenumbers in OW (cm^−1^)	Peak assignment in the common range
1,000–1,015	■	1,001, 1,004, 1,008, 1,012	C-C stretching (carotenoids/lipid backbone) (18)
1,100–1,172	1,143	1,169, 1,172	C-C stretching (carotenoids) C-C stretching + CH_3_ rocking (lipids/carotenoids)
1,628–1,682	1,621, 1,624, 1,628, 1,631, 1,634, 1,678, 1,682	1,631, 1,645, 1,662, 1,665, 1,668, 1,675, 1,678	C=C stretching (unsaturated lipids) Amide I (proteins)
1742–1756	1,742, 1,756		C=O ester stretching (triglycerides, phospholipids) (13)
2,859–3,000	2,859, 2,862, 2,865, 2,873, 2,887, 2,890, 2,896, 2,996	2,821, 2,827, 2,830, 2,833, 2,836, 2,839, 2,844, 2,847, 2,850, 2,853, 2,862, 2,865, 2,867, 2,870, 2,873	CH_2_ symmetric (2,840) & asymmetric (2,880) stretching (lipid acyl chains) (13) CH_2_/CH_3_ stretching (13)
3,000–3,050	3,010, 3,012, 3,015, 3,018, 3,021, 3,024		= C-H stretch (unsaturated lipids,*cis* double bonds) (18)

## Conclusion

In summary, this study demonstrates the effectiveness of integrating advanced multivariate analysis—specifically the combination of Random Forest feature selection and UMAP visualization—for the nuanced classification of Raman spectral data from breast adipocyte subtypes. By correcting carotenoid interference and identifying discriminant wavenumbers, this approach revealed subtle, yet meaningful, biochemical distinctions between cancer-associated adipocytes (CAA) and normal adipocytes (NA), as well as highlighted BMI-related variations within these populations. Notably, the identification of key discriminant regions, such as those corresponding to lipid and protein-associated vibrations, provides deeper insight into the molecular underpinnings of adipocyte heterogeneity and their potential roles in the tumor microenvironment. While the study offers promising evidence for the application of this integrative analytical pipeline, larger cohorts and further validation are necessary to generalize these findings and to fully unravel the clinical implications. Future efforts should therefore focus on expanding sample diversity, refining spectral preprocessing, and correlating spectral biomarkers with functional and pathological endpoints. Ultimately, such approaches hold the potential to contribute to precision diagnostics and to advance our understanding of the dynamic crosstalk between adipose tissue and breast cancer progression.

## Data Availability

Data will be made available on request.

## Supplemental Data

This article contains supplemental data.

## Conflict of Interest

The authors declare that they have not conflict of interest with the contents of this article.

## Figures and Tables

**Supplemental Figure S1:**